# Improving the Supercritical CO_2_ Foaming of Polypropylene by the Addition of Fluoroelastomer as a Nucleation Agent

**DOI:** 10.3390/polym11020226

**Published:** 2019-02-01

**Authors:** Chenguang Yang, Quan Zhao, Zhe Xing, Wenli Zhang, Maojiang Zhang, Hairong Tan, Jixiang Wang, Guozhong Wu

**Affiliations:** 1 Shanghai Institute of Applied Physics, Chinese Academy of Sciences, Jialuo Road 2019, Jiading, Shanghai 201800, China; yangchenguang@sinap.ac.cn (C.Y.); zhaoquan@htkjbattery.com (Q.Z.); xingzhe@sinap.ac.cn (Z.X.); zhangwenli@sinap.ac.cn (W.Z.); zhangmaojiang@sinap.ac.cn (M.Z.); tanhairong@sinap.ac.cn (H.T.); wangjixiang@sinap.ac.cn (J.W.); 2 University of China Academy of Sciences, Beijing 100049, China; 3 School of Physical Science and Technology, ShanghaiTech University, Haike Road 100, Pudong, Shanghai 201210, China

**Keywords:** polypropylene, fluoelastomer, scCO_2_ foaming, heterogeneous nucleation

## Abstract

In this study, a small amount of fluoroelastomer (FKM) was used as a nucleating agent to prepare well-defined microporous PP foam by supercritical CO_2_. It was observed that solid FKM was present as the nanoscale independent phase in PP matrix and the FKM could induce a mass of CO_2_ aggregation, which significantly enhanced the diffusion rate of CO_2_ in PP. The resultant PP/FKM foams exhibited much smaller cell size (~24 μm), and more than 16 times cell density (3.2 × 10^8^ cells/cm^3^) as well as a much more uniform cell size distribution. PP/FKM foams possessed major concurrent enhancement in their tensile stress and compressive stress compared to neat PP foam. We believe that the added FKM played a key role in enhancing the heterogeneous nucleation, combined with the change of local strain in the multiple-phase system, which was responsible for the considerably improved cell morphology of PP foaming. This work provides a deep understanding of the scCO_2_ foaming behavior of PP in the presence of FKM.

## 1. Introduction

As a widely investigated commercial polymer, polypropylene (PP) foam has numerous desirable and beneficial properties, such as good chemical-resistance, outstanding mechanical properties, low electrical conductivity, low cost and a unique porous honeycomb structure [1,2,3,4]. PP foams have wide range of many industrial applications in the fields of packaging, aerospace, automobiles, acoustic absorbent, dielectric materials, energy storage materials, thermal insulators, as well as tissue engineering [1,2,5,6,7,8,9]. However, due to their very low melt strength and high crystallinity, the fabrication of linear PP foams is not successful [10,11,12,13]. Consequently, the resultant neat PP foam usually exhibits large cell diameter, low cell density, and poor mechanical properties.

To improve the melt strength, considerable efforts have been made to optimize the process of PP foaming, enhance PP foam ability as well as improve cellular structure [12,14,15,16,17,18,19], such as long-chain branching, crosslinking [11,16,20,21], polymer blending [12,22], and compounding [23,24]. In recent years, it was found that nano-materials such as carbon nanotubes, carbon nanofibers, and graphene added in PP could enhance heterogeneous nucleation to increase cell density, reduce cell size, improve cell size uniformity, and at the same time reinforce the PP matrix [12,18,25,26,27,28]. But the cost of these nanoparticles is expensive, so it is difficult to use them for the high-volume production of PP foams [12]. Moreover, the foaming behavior of polymer is greatly influenced by the solubility of CO_2_, which determines the cellular structure, expansion ratio, and crystallization parameters of the resultant foams [29,30,31]. In addition, the use of scCO_2_ can decrease the melt viscosity of the polymer owing to the strong plasticizing effect of the dissolved CO_2_ and thus improve the processability of polymers [12,18,29,30,32].

Thermoplastic fluoroelastomer (FKM) possesses outstanding chemical-resistant, high melt point, excellent weather resistance as well as flame retardant properties. In particular, good affinity and solubility between fluorine compunds and carbon dioxide were found [33,34]. However, PP foaming by scCO_2_ has not been investigated in the presence of FKM. Herein, a small amount of FKM was applied as the nucleating agent to improve scCO_2_ foaming behavior of PP. The results showed that enhanced heterogeneous nucleation and increased foaming ability were obtained in the presence of FKM. The saturated mixed phases (PP/FKM/CO_2_) are like an “island model”, and the existence of FKM can increase the number of the heterogeneous nucleation sites during the foaming process. The obtained PP/FKM foam possessed large cell density, small cell size, uniform cell size distribution as well as an excellent expansion ratio. In addition, the resultant PP/FKM foams endowed unusual tensile and compressive strength across a wide foaming pressure range. Furthermore, the foaming parameters of PP/FKM including saturation pressure and saturation time were also investigated in this work.

## 2. Materials and Methods

### 2.1. Materials

Random polypropylene (Sep-540) with a density of 0.89 g/cm^3^ and a melt flow rate (MFR) of 7.0 g/10 min was purchased from LOTTE Chemical Co. (Jiaxing, China) Fluoroelastomer (FKM 246) with a density of 1.86 g/cm^3^ was supplied by Sinopec Shanghai Chemical Co. (Shanghai, China). CO_2_ with a purity of 99.95% was used as a foaming agent.

### 2.2. Sample Preparation

The PP pellets and FKM were dried at 60 °C for 4 h before they were used. A series of mixtures with FKM contents of 0.5, 1.0, and 2.0 wt %, were prepared at 240 °C using a two-screw extruder (Thermo Haake PolyDrive 7, Shanghai, China). The extruded strands were cooled in water and pelletized with a strand cutter. PP/FKM sheets with a thickness of 1 mm were obtained by hot-pressing under the conditions of 190 °C and 20 MPa. For comparison, a neat PP sheet was also prepared. The samples were denoted as PP/FKM(0.5), PP/FKM(1.0), and PP/FKM(2.0), respectively. The characteristic parameters of the mixtures are shown in Table 1.

### 2.3. Foaming Process

PP sheet samples were placed in a high pressure autoclave, and the autoclave was pressurized with CO_2_ using a plunger metering pump; the parameters of the foaming device have been described in the previous literature [11,12,16,18,19,21]. The system was kept at the pre-set temperature and pressure for 1 h. Then, the autoclave was vented in less than 10 s. Finally, the samples were removed from the autoclave and cooled to room temperature.

### 2.4. Sample Characterization

A differential scanning calorimeter (DSC, NETZSCH STA 449 F3 Jupiter, Shanghai, China) was used to scan the melting transitions of the specimens in aluminum crucibles. The specimens were first heated from 25 to 250 °C at 10 °C/min under an argon flow (20 mL/min), then cooled to 30 °C at 10 °C/min under an argon flow (20 mL/min), and again heated to 250 °C under the same conditions. The first heating was performed to eliminate the thermal history of the specimens. The crystallization parameters of the specimens were obtained from the software Proteus-6 (NETZSCH, Shanghai, China). The crystallinities were calculated using Equation (1).

(1) $$X_{c}\left(\%\right)=\frac{{\Delta H}_{f}}{{\Delta H}_{f0}}\times 100$$

where ${\Delta H}_{f}$ is the melting enthalpy measured in the heating process, and ${\Delta H}_{f0}$ is the theoretical enthalpy of 100% crystalline PP, 207.1 J/g [35].

The micro-morphologies of the unfoamed and foamed specimens were observed using a scanning electron microscope (SEM, Zeiss MERLIN Compact 14184, Shanghai, China). Samples were immersed in liquid nitrogen for 2 min, then fractured, mounted on stubs, and sputter-coated with gold.

### 2.5. Morphological Observation of the Foams

Image Pro-Plus software 6.0 (Media Cybernetics, Rockvill, MD, USA) was used to analyze the SEM images. The average cell size D of the cells in the micrographs was calculated using Equation (2).

(2) $$D=\frac{\sum d_{i}n_{i}}{\sum n_{i}}$$

where $n_{i}$ is the number of cells with a perimeter-equivalent diameter of $d_{i}$. To ensure the accuracy of the average cell size measurement, i is greater than 200.

The volume expansion ratio of each foam was calculated as the ratio of the density of the solid PP, $\rho_{s}$, to the measured density of the foam, $\rho_{f}$. The foam density ($\rho_{f}$) was determined from Archimedes’ law by weighing the PP foam in water with a sinker using an electronic analytical balance (HANG-PING FA2104) and the foam density was calculated using Equation (3).

(3) $$\rho_{f}=\left(\frac{a}{a+b-c}\right)\rho_{w}$$

where *a*, *b*, and *c* are the weights of the specimen in air without the sinker, the totally immersed sinker, and the specimen immersed in water with the sinker, respectively, and $\rho_{w}$ is the density of water.

The volume expansion ratio ($V_{f}$) was calculated using Equation (4).

(4) $$V_{f}=\frac{\rho_{s}}{\rho_{f}}$$

where $\rho_{s}$ and $\rho_{f}$ are the density of un-foamed and foamed samples, respectively.

The porosity $P_{f}$ is related to the density of the PP foam $\rho_{f}$ and the un-foamed PP $\rho_{s}$, which was calculated using Equation (5).

(5) $$P_{f}(\%)=\left(1-\frac{\rho_{s}}{\rho_{f}}\right)\times 100$$

The cell density ($N_{0}$) was calculated using Equation (6).

(6) N0=(nA)32Vf

where n and A are the number of cells in the SEM image and the area of the image (cm^2^), respectively.

### 2.6. Mechanical Properties

The tensile strength of the un-foamed and foamed specimens was measured using a universal testing machine (5943, Instron, Shanghai, China). The foams were cut into 2 mm × 4 mm × 25 mm pieces. All the specimens were measured in accordance with ASTM D-638 at a room temperature of 23 °C. The compression strength of the resultant foams was measured using an MTS universal microtester (Jinan zhongchuang testing machine technology Co. LTD, Jinan, China) equipped with a 50 N load cell. The side lengths of 6 mm of cubic specimens cut from the foamed samples were employed for compression tests; the speed was 1 mm/min, and more than five data points were measured for each sample under the same conditions.

## 3. Results and Discussion

### 3.1. Microscopic Structure of PP/FKM Blends

**Figure 1:**
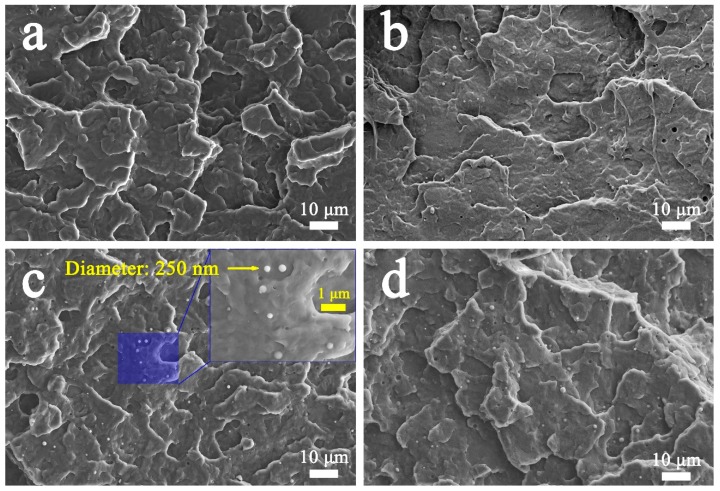
SEM images of fractured surfaces of (**a**) neat PP, (**b**) PP/FKM(0.5), (**c**) PP/FKM(1.0), and (**d**) PP/FKM(2.0) samples.

PP/FKM mixtures with various FKM contents were mixed by an extrusion system. To understand the dispersion of FKM in the PP phase, we analyzed the SEM micrographs of PP/FKM samples. Figure 1 shows the fractured surface images of the resultant neat PP and PP/FKM mixtures. It can be easily seen that FKM has an excellent dispersion in PP matrix. The size of the formation dispersion phase of FKM was about 250 nm in the PP phase and it could be clearly seen that the sizes of FKM particles in PP matrix were all at the nanoscale level. According to the literature [33,34], the FKM phase is still in a solid state at the foaming temperature (152 °C), so the FKM may play a role as the nucleating agent in forming the cellular structure of PP during the foaming process.

### 3.2. Morphologies and Properties of PP/FKM Foams

**Figure 2:**
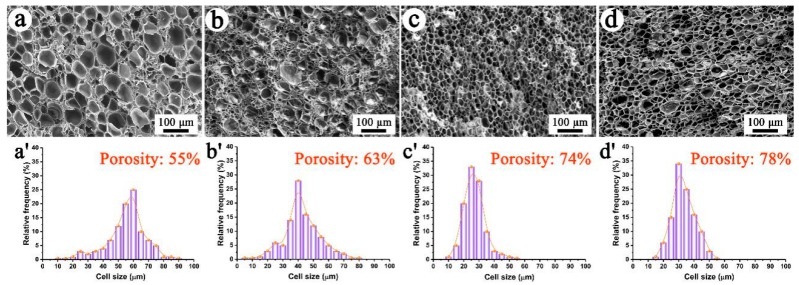
SEM images and cell size distributions of foams (**a**) neat PP, (**b**) PP/FKM(0.5), (**c**) PP/FKM(1.0), and (**d**) PP/FKM(2.0), all prepared at 152 °C and 20 MPa.

The cell morphologies of neat PP, PP/FKM(0.5), PP/FKM(1.0), and PP/FKM(2.0) foams prepared at 152 °C and 20 MPa are shown in Figure 2. The cell size declined as the loading of FKM increased to 1.0 wt %. Cracked and consolidated cells appeared, and the cell continuity was poor in the neat PP foam. The PP/FKM foams exhibited a different cellular structure with different FKM contents. The cell size distributions of different foams are shown in Figure 2. PP/FKM foams possessed narrower cell distributions than those of the neat PP foam. In particular, PP/FKM(1.0) and PP/FKM(2.0) foams exhibited much more uniform cell size distribution. Moreover, increased porosities were obtained as the loading of FKM increasing, which indicated a much better foaming ability of PP. These results implied that the enhanced diffusion rate and increased solubility of CO_2_ were obtained in the presence of FKM, resulting in a large porosity, which was also found in the previous studies on fluorinated ethylene propylene copolymer (FEP) foaming by scCO_2_ [35,36]. Furthermore, the independent solid-state FKM phase in the PP matrix may significantly increase the heterogeneous nucleation site in the foaming process.

**Figure 3:**
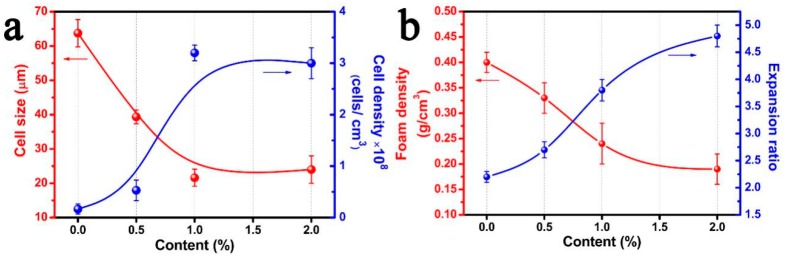
(**a**) Cell diameter and cell density, (**b**) Foam density and expansion ratio of neat PP, PP/FKM(0.5), PP/FKM(1.0), and PP/FKM(2.0) foams.

**Figure 4:**
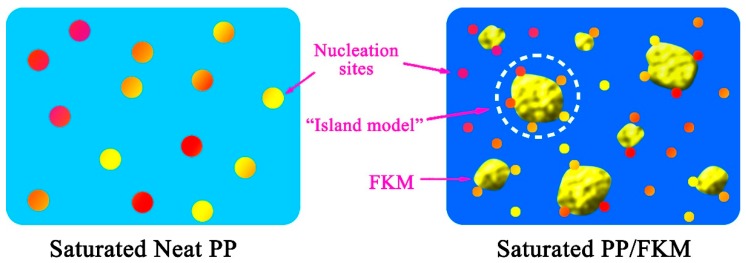
Schematic diagram showing the nucleation mechanism of the inner region of neat PP and PP/FKM samples. For clarity, the symbols are not proportional to the real size.

The average cell diameter and cell density of the cellular structure of the neat PP and PP/FKM(0.5), PP/FKM(1.0), and PP/FKM(2.0) foams are summarized in Figure 3a. The cell size of PP foams decreased from 65 to 23 μm as the loading of FKM increased from 0 to 1.0 wt % and the cell density increased significantly compared to neat PP foam, by more than 16 times. The foam density and expansion ratio of foamed samples are shown in Figure 3b. The foam density declined as the content of FKM increased from 0 to 2.0 wt %, in agreement with the results in Figure 2. The existence of FKM led to a higher diffusion rate and solubility of CO_2_ in the melt PP matrix, and enough CO_2_ could support cell growth for a long time [35,36]. According to “Heterogeneous Nucleation Theory”, the formed nanoscale solid FKM phase in the PP matrix can act as the nucleating agent; it is vividly shown in Figure 4. It is known that foaming is a rapid process for cells growing in a few seconds, which depends on the thermophysical and rheological properties of PP/CO_2_ mixtures, and this process is related to the change of temperature, pressure, and local stress, etc. In the multiple phase system, the existence of the FKM phase induces a mass of CO_2_ aggregation, which is similar to an “island”. During the process of release pressure, it was easy to cause the change of local stress around the “island”, and induce a large number of nucleation sites, which greatly increased the nucleation rate. Furthermore, a large number of cell sites, caused by heterogeneous nucleation, competed for the limited CO_2_, which restricted the cell growth [37]. Additionally, the suitable foaming conditions of FKM were about 230 °C and 30 MPa [35]. A small amount of CO_2_ might dissolve into the FKM phase in the saturation process, so it might also enhance the foaming ability of PP/FKM samples.

**Figure 5:**
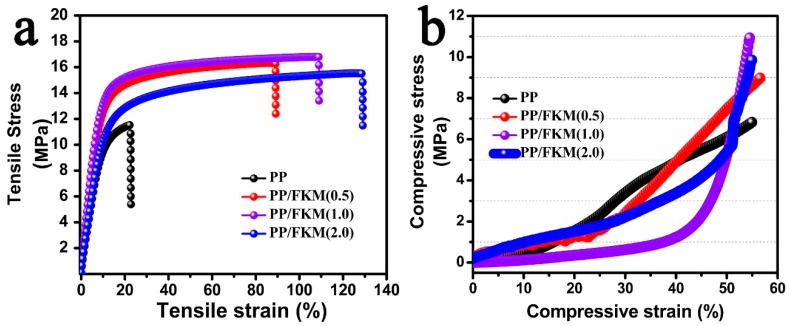
Mechanical properties of neat PP and PP/FKM foams: (**a**) tensile strength and (**b**) compressive strength.

The mechanical properties of the resultant PP foam are the important evaluation parameters for potential industrial applications. The tensile stress-strain and compressive stress-strain curves of neat PP and PP/FKM foams are shown in Figure 5. The PP/FKM foams possess excellent stress and strain compared to neat PP foam. The tensile stress increased to more than 15 MPa and the tensile strain of PP/FKM foam reached 110%. The compressive strength results showed that PP/FKM exhibited higher stress than neat PP foam. The obtained outstanding mechanical properties were ascribed to the well-defined cellular structure and high continuous polygonal cell morphology of PP/FKM(1.0) foam [10,12,18,19]. All these clear results signified the key role of FKM in preparing fine PP foam with excellent mechanical properties, which indicated promising engineering applications.

### 3.3. Effects of Foaming Pressure on the Foaming Behavior of PP/FKM(1.0)

**Figure 6:**
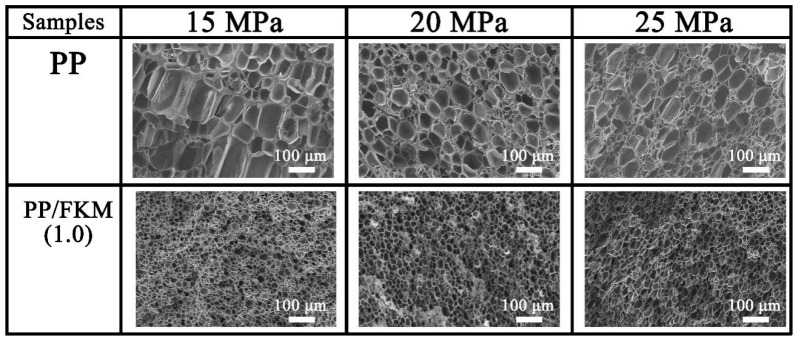
SEM images of PP/FKM(1.0) foams prepared at 152 °C and different pressures: 15, 20, and 25 MPa.

Figure 6 shows the effects of different saturation pressures on the cell morphologies of PP/FKM(1.0) samples at 152 °C. All the specimens foamed regardless of the pressures (15, 20, and 25 MPa). However, there was large difference between the cellular structures of neat PP foams prepared at different pressures. There were non-foaming regions and non-uniform cell size in the neat PP foam prepared at 15 MPa and this was caused by insufficient swelling of CO_2_. The resultant PP/FKM(1.0) foams exhibited good cellular structures at different saturation pressures.

**Figure 7:**
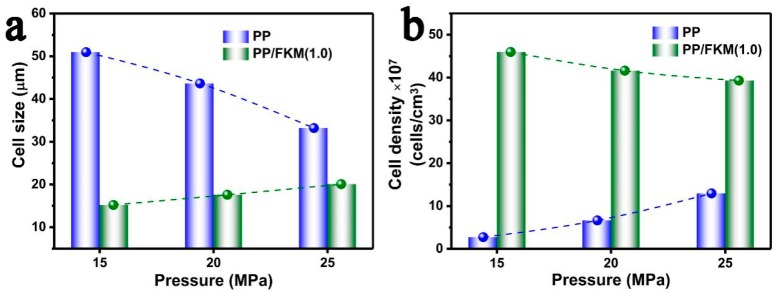
(**a**) Cell size and (**b**) cell density of PP/FKM(1.0) foams prepared at 152 °C and different saturation pressures.

The cell diameter and cell density of neat PP and PP/FKM(1.0) foams are summarized in Figure 7. It can be seen that the PP/FKM(1.0) foams showed small average cell size and high cell density. In addition, the cells of PP/FKM(1.0) foams almost all exhibited good continuity at different pressures, which indicated good mechanical properties. In general, cell size increased as the pressure increased for the enhanced expansion force of CO_2_ against the cell wall. We believe that the existence of the nanoscale FKM phase increased the solubility of CO_2_ around the FKM “island” in the PP matrix, improving the foaming ability of PP/FKM.

### 3.4. Effects of Saturation Time on the Foaming Behavior of PP/FKM(1.0)

**Figure 8:**
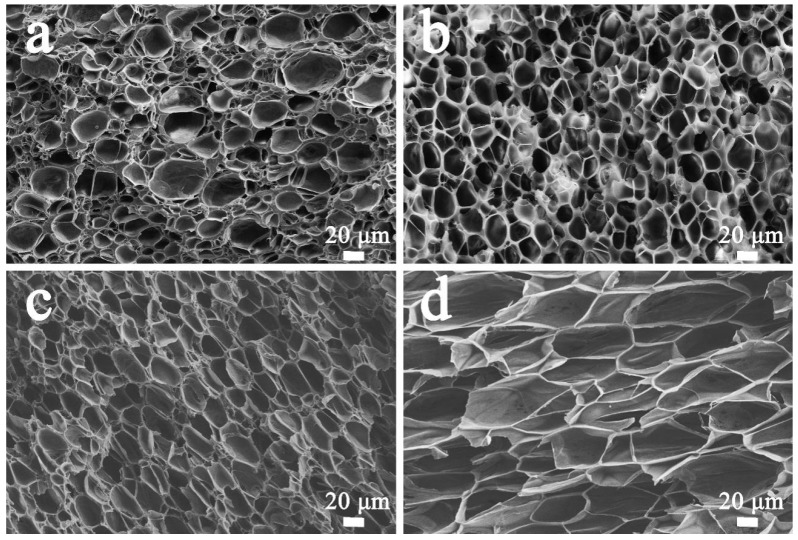
SEM images of PP/FKM foams prepared at 152 °C and 20 MPa with different saturation times, (**a**) 30 min, (**b**) 60 min, (**c**) 90 min, and (**d**) 120 min.

From the previous discussion, the porosities of PP/FKM foams increased as the loading of FKM increased and we ascribed it to the increased solubility of CO_2_ in the PP matrix. It is known that the solubility of CO_2_ is also affected by the saturation time. Consequently, the influence of different saturation times on the cellular structures of PP/FKM(1.0) foams was also studied. It could be seen that the foaming ability of PP/FKM(1.0) was significantly enhanced as the saturation time increased, as shown in Figure 8. Some non-foamed regions could be clearly seen in Figure 8a. The reason was that the melt PP matrix was not fully swelled by CO_2_ in 30 min, so it was inclined to form a non-uniform distribution of cell size. More CO_2_ dissolved into PP as the saturation time increased, which improved the foaming ability of PP. As CO_2_ continually dissolved into the melt PP, the increased expansion force of CO_2_ further supported the cell growth, resulting in a larger cell size.

**Figure 9:**
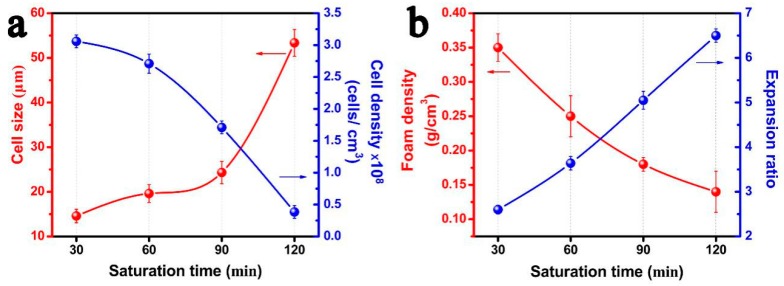
(**a**) Average cell diameter and cell density, (**b**) Foam density and expansion ratio of PP/FKM(1.0) foams prepared at different saturation times (30 min, 60 min, 90 min, and 120 min).

**Figure 10:**
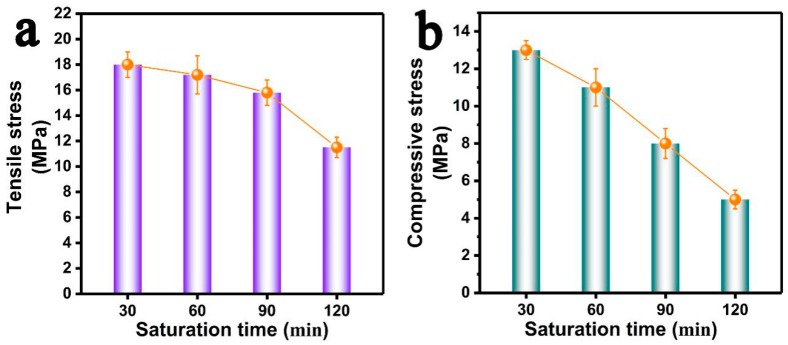
(**a**) Tensile stress and (**b**) compressive stress of PP/FKM foams prepared at different saturation times (30 min, 60 min, 90 min, and 120 min).

Figure 9 summarizes the parameters of the cellular structure of PP/FKM(1.0) foams as a function of saturation time. The cell size increased and the cell density decreased as the saturation time increased from 30 to 120 min. The foam density of resultant PP/FKM(1.0) foams showed the declining phenomenon as the saturation time increased, which indicated an increasing porosity and expansion ratio of the foams. These results signified that the solubility of CO_2_ was further increased as the saturation time increased, and a large amount of CO_2_ supported cell growth. The changes of the tensile stress and compressive stress of the foams prepared at different saturation times are shown in Figure 10. It is observed that the mechanical properties decrease as the saturation time increases. A well-defined cellular structure with uniform cell size distribution and good cell continuity, often exhibited good elasticity during the tensile and compressive process, resulted in unusual properties [38,39]. These results are consistent with the conclusion in the previous studies [7,10,12,38,39].

## 4. Conclusions

In this study, microcellular PP foam with fine cellular structure was fabricated in the presence of FKM by scCO_2_ foaming. It was found that the nanoscale solid FKM phase induced a mass of CO_2_ aggregation, which was similar to the “island model”, and the FKM could greatly enhance the heterogeneous nucleation as the nucleation agent during the foaming process. The resultant PP/FKM foams exhibited smaller cell size, and more than 16 times higher cell density compared to neat PP foam. PP/FKM foams possessed a higher tensile and compressive stress compared to neat PP foam. The results also showed that FKM significantly improved the cell morphology parameters of PP/FKM foams in a large foaming pressure window. Finally, the obtained PP foams with various performance parameters could be easily controlled by changing the FKM content, foaming temperature and saturation time.

## Figures and Tables

**Table 1:** Characteristic parameters of neat PP and PP/FKM mixtures.

Samples	PP	PP/FKM(0.5)	PP/FKM(1.0)	PP/FKM(2.0)
T_m_/°C	165.4	165.9	166.4	167.3
X_C_/%	38.2	38.8	39.7	40.4
